# LED Curing Lights and Temperature Changes in Different Tooth Sites

**DOI:** 10.1155/2016/1894672

**Published:** 2016-04-18

**Authors:** E. Armellin, G. Bovesecchi, P. Coppa, G. Pasquantonio, L. Cerroni

**Affiliations:** ^1^Department of Clinical Science and Translational Medicine, University of Rome Tor Vergata, Via Montpellier 1, 00133 Rome, Italy; ^2^Faculty of Engineering, University of Rome Tor Vergata, Via del Politecnico 1, 00133 Rome, Italy

## Abstract

*Objectives.* The aim of this* in vitro* study was to assess thermal changes on tooth tissues during light exposure using two different LED curing units. The hypothesis was that no temperature increase could be detected within the dental pulp during polymerization irrespective of the use of a composite resin or a light-curing unit.* Methods.* Caries-free human first molars were selected, pulp residues were removed after root resection, and four calibrated type-J thermocouples were positioned. Two LED lamps were tested; temperature measurements were made on intact teeth and on the same tooth during curing of composite restorations. The data was analyzed by one-way analysis of variance (ANOVA), Wilcoxon test, Kruskal-Wallis test, and Pearson's *χ* ^2^. After ANOVA, the Bonferroni multiple comparison test was performed.* Results.* Polymerization data analysis showed that in the pulp chamber temperature increase was higher than that without resin. Starlight PRO, in the same condition of Valo lamp, showed a lower temperature increase in pre- and intrapolymerization. A control group (without composite resin) was evaluated.* Significance.* Temperature increase during resin curing is a function of the rate of polymerization, due to the exothermic polymerization reaction, the energy from the light unit, and time of exposure.

## 1. Introduction

The temperature of the tooth pulp chamber (from 34 to 35°C) can be influenced by different dental procedures. Temperature increases could be due to use of high-speed instruments and exothermal reaction of provisional resin-based materials or composite resin polymerization reaction which could damage the pulp tissue [1]. Postoperative sensitivity, pain, or even pulp necrosis may represent the possible adverse evolution closely related to the heating [2, 3].

The use of a blue-light-emitting diode (LED) as an alternative method of light curing was suggested in 1995 to overcome the problem of the quartz-tungsten-halogen lights such as higher operating temperature, reduction in efficacy over time, and insufficient physical properties [4]. LED curing lights were reported to cure resin-based composites with resulting properties similar to those obtained with standard halogen light [5, 6]. The potential for reduced irradiation time is limited by the need to maintain Vickers hardness values as high as possible [7]. In fact, a low-energy density with an increased duration of exposure may be more advisable than a high-power intensity for the cure of composite resins at a depth of 3 mm [8].

The potential risk of pulpal injury during composite polymerization is increased with new light-curing units with higher-energy output compared with previous generation [9], which may result in increased heat transmitted to the pulp [10]. Since the dental pulp is a low-compliance system which does not respond well to increased temperature, the heat emitted during the polymerization of composite resins may cause significant temperature increases within the pulp chamber, finally harming the dental pulp connective tissue [11].

Pulp tissue consists of a relatively large amount of tissue with a small vascular terminal circulation (with no collateral supply) encased within hard dentinal walls [12]. Nevertheless, pulp cells may survive such injuries, possibly due to the increased synthesis of heat-shock proteins [13, 14] and the remaining dentin thickness [15, 16]. The mechanism that leads to pulpal damage includes coagulation, expansion of the fluid in the dentinal tubules, vascular damage, and tissue necrosis.

The thermal behavior of teeth is mainly a heat conduction process coupled with the tooth's physiological processes (dentinal fluid flow and pulpal blood flow). The thermophysical properties of teeth vary between different layers (enamel and dentin) and depend on their microstructures. The thermal conductivity of human dentin decreases with the increasing volume fraction of dentin tubules: the higher the degree of mineralization, the greater the increase in the pulp chamber [16]. The flow of dentinal fluid within the dentin tubules when heated can also enhance heat conduction within the pulp, but when the curing lights are turned off, the decrease in pulp temperature is more pronounced when the flow rate is higher [17]. An increase of the intrapulpal temperature exceeding 42.5°C can result in structurally irreversible damage in the pulp tissue. During the polymerization of light-activated resin composite, the exothermic reaction process and the energy absorption during irradiation can cause an important temperature increase in the pulp chamber, which has been quantified as ranging from 2.9 to 7.8°C [18, 19]. Thus, it is possible that the radiation in the wavelength in the peak activation range (from 440 to 500 nm) of camphorquinone contributes to heating of the composite. Reducing the irradiation spot size to concentrate the curing irradiation at the centre of the composite resulted in a higher curing ability (increasing the scraping depth), while concentrating the energy toward the pulp.

In this* in vitro* study, we analyzed the most important points connected with the increase of temperature in the pulp chamber, with particular attention given to polymerization with different kinds of LEDs. The hypothesis of this study is that there are differences in behavior between lamps that have more spectra than the lamps that have only one.

The purpose of this study was to evaluate thermal changes in the tooth structures induced by two different LED curing units, with and without resin composite polymerization.

## 2. Materials and Methods

Fifteen caries-free human first molars were stored in 0.5% chloramine in water at 4°C and used within 1 month after extraction, with the approval of the Ethics in Research Committee of the Centre of Health Sciences of the University of Rome “Tor Vergata,” Rome, Italy.

Pulp residues were removed after root resection; then specimens were randomly assigned to 3 groups (*N* = 3; tooth intact, without composite, and with composite). The experimental set-up was designed by Keithley: model 2700 with a 7700 card, data acquisition system (DAS), and a set of type-J thermocouples (range, from −40°C to +750°C; sensitivity, 50 *μ*V/°C, with a precision of ±0.1°C) with a cold junction in ice. The DAS allowed for the acquisition of signals with a resolution of 6.5 digits and a scanning range between a given and the next of 0.128 s.

**Figure 1:**
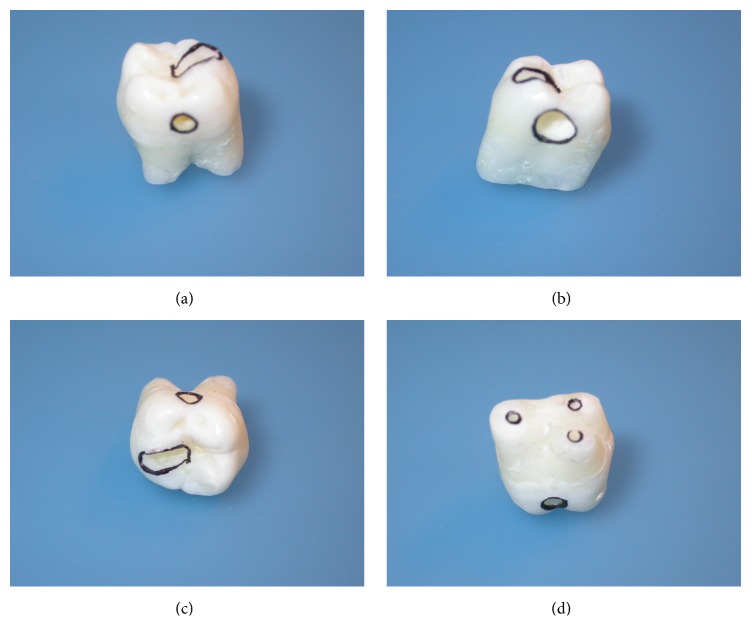
Tooth preparation: (a) mesial view with the thermocouple box in the middle position; (b) distal view with the obturation box; (c) occlusal view with the thermocouple box in the occlusal position; and (d) root view showing the hole for the thermocouple in the pulp chamber.

**Figure 2:**
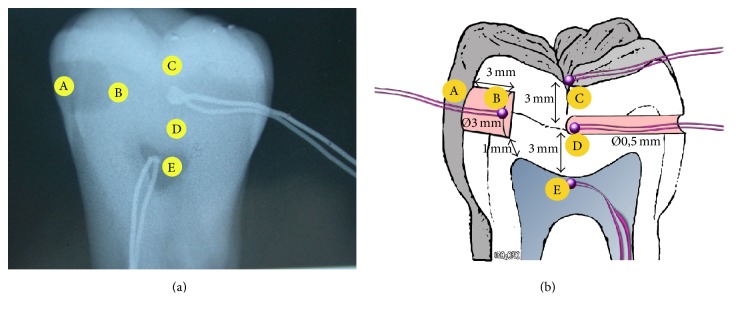
X-ray of tooth showing the thermocouple in position + schematic representation.

Tooth preparations for housing the thermocouples were prepared by means of a cylindrical diamond bur drill (Sweden & Martina SPA, Via Veneto 10, 35020 Due Carrare (PD), Italia, model 837 blue, 7.0 mm, iso diameter 0.16) and the customized thermocouples with a thin-coated wire (0.5 mm in diameter) capable of rapid response were located in the tooth. Thermocouple A (Figure 1) was placed on the distal surface of the crown, for measurement of the temperature increase in the area directly exposed to light (*d* = 0). Thermocouple C (Figure 1) was positioned on the occlusal surface 5 mm from thermocouple A, and thermocouple D was inserted into a box on the mesial side, 6 mm from the distal surface. After root resection, irrigation of the endodontic space with sodium hypochlorite was performed; then thermocouple E was placed into the pulp chamber filled with ultrasound gel (Eco Supergel; Ceracarta, Forlì, Italy). During all the measurements the tooth was immersed in ultrasound gel. On the same specimen, after measurements with the LED lamp, a box of 3 mm diameter and 3 mm depth was prepared at the point where thermocouple A was previously located, 1 mm away from the pulp chamber. Thermocouple B was placed at the bottom of the box. A radiograph was taken to confirm the position of the thermocouple (Figures 1 and 2).

A microhybrid resin composite (Enamel Plus HFO A2; Micerium S.p.A., Avegno [GE], Italy) was used for restorations. In this work bounding was not used to avoid uncontrollable variables.

Two LED lamps were selected and tested: VALO (Ultradent Products, South Jordan, UT, USA), tested at a light intensity of 1000 mW/cm^2^ (for 20 s) or 3200 mW/cm^2^ (for 3 s); Starlight PRO (Mectron S.p.A., Carasco [GE], Italy), tested at a light intensity of 1000 mW/cm^2^ (for 20 s).

Absolute intensity of the two lamps was performed with a bolometer (Coherent LM10, Santa Clara, CA, USA) to assess the effective lamp output, and the effective lamp tip diameter was measured in order to evaluate the real energy emitted by the lamp. Results are summarized in Table 1.

**Figure 3:**
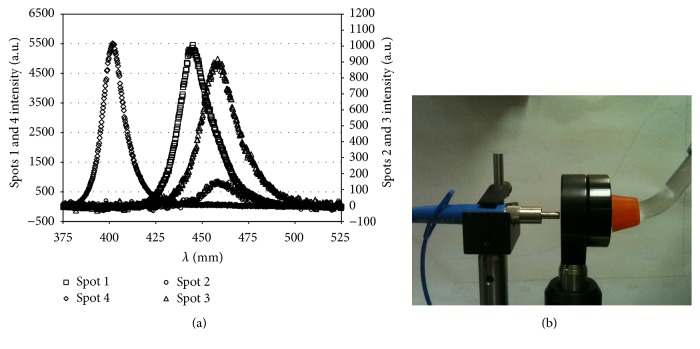
(a) Spectral distribution of the light emitted by 4 sources of the LED lamp. (b) Spectrophotometer.

VALO lamp was also tested with a spectrophotometer (HR4000, Ocean Optics, Dunedin, FL, USA) to measure the wavelength response of the 4 sources of LED lamp (Figure 3).

Measurements of thermal change (Δ*T*) were made on each intact tooth before box preparation and on the same tooth during the polymerization of resin composite restorations (Figure 3). Investigated variables were polymerization mode (differences between the three groups) and role of the restoration. Maximum temperature increases for the three groups of specimens were analyzed by one-way analysis of variance (ANOVA), the Wilcoxon test, the Kruskal-Wallis test, and Pearson's *χ* ^2^. After ANOVA, the Bonferroni multiple comparison test was performed.

## 3. Results

**Figure 4:**
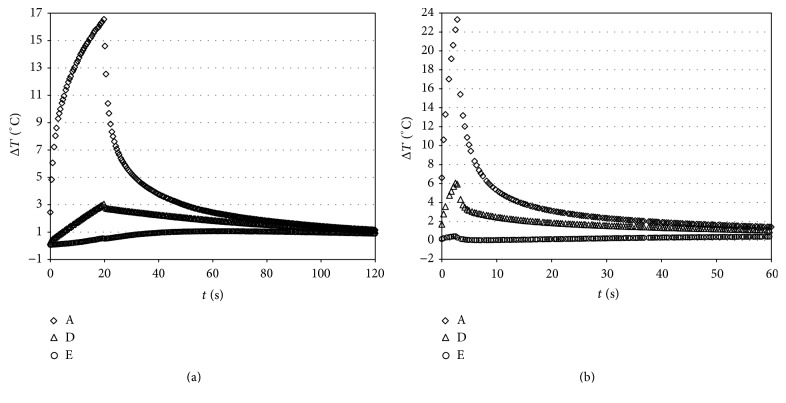
Characteristic Δ*T* (°C) versus time curves caused by irradiation with VALO (Ultradent), tested at a light intensity of 1000 (for 20 s) (a) or 3200 (for 3 s) mW/cm^2^ (b). A (3 mm from light source); D (mesial); E (pulp chamber) before box preparation.

**Figure 5:**
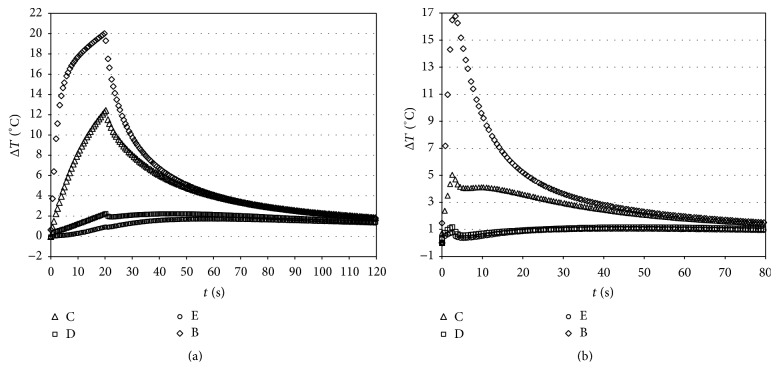
Characteristic Δ*T* (°C) versus time curves caused by irradiation with VALO (Ultradent), tested at a light intensity of 1000 (for 20 s) (a) or 3200 (for 3 s) mW/cm^2^ (b) during composite polymerization. B (bottom of the box); C (occlusal surface); D (mesial); E (pulp chamber).

Figures 4 and 5 report Δ*T* versus time curves for VALO tested at a light intensity of 1000 mW/cm^2^ (for 20 s; Group 1) or 3200 mW/cm^2^ (for 3 s; Group 2), respectively, before and after polymerization.

Three different sections can be identified in the Δ*T* curves. In the first section, the temperature remains constant, depending on the time needed to reach thermal equilibrium with the surrounding environment of the specimen.

Means and standard deviations of maximum temperature increase resulting from different tooth sites before and during composite polymerization with VALO or Starlight PRO are reported in Tables 2 and 3. Maximum temperature increase with VALO at a light intensity of 1000 mW/cm^2^ at 1 mm from the occlusal surface thermocouple was 21.78 ± 4.69°C (Group 1), with VALO at 3200 mW/cm^2^ was 34.66 ± 4.93°C (Group 2), and with Starlight PRO at 1000 mW/cm^2^ was 17.88 ± 2.36°C (Group 3). The Starlight PRO showed little increase of temperature relative to the other mode of curing (*p* ≤ 0.01).

When the composite was light-cured, the temperature values increased rapidly, reaching a plateau in 0.7–1 s. Thermal flux generated by monomer conversion was added to light-curing heat.

Analysis of the data obtained during composite polymerization with VALO at a light intensity of 1000 mW/cm^2^ (Group 1, Figure 5) showed that, in the pulp chamber, the temperature increase was greater than those obtained without composite. Moreover, the temperature in the pulp chamber was higher than at other thermocouple positions.

**Figure 6:**
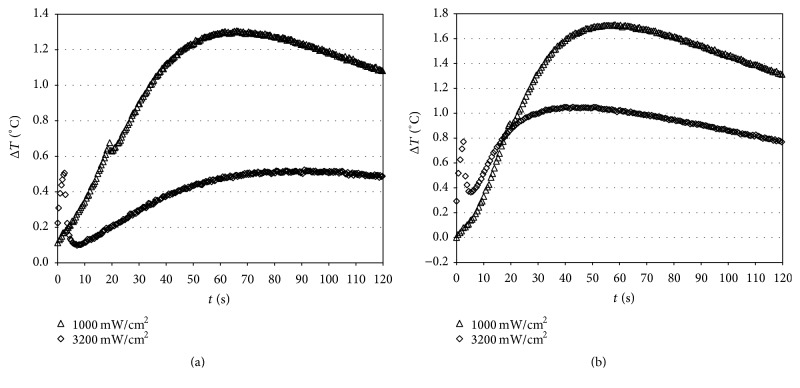
Characteristic pulp chamber Δ*T* (°C) versus time curves caused by irradiation with VALO (Ultradent), tested at a light intensity of 1000 (for 20 s) or 3200 (for 3 s) mW/cm^2^ without (a) and during composite polymerization (b).

It is clear that prolonged exposure for 20 s resulted in higher elevations of temperature with respect to light intensity of 3200 mW/cm^2^ for 3 s, although the power was lower (Figure 6). In fact, the temperature diffusion was higher with VALO at 1000 mW/cm^2^ (Group 1), determining a temperature increase in various parts of the tooth of approximately 2.75 to 3.21°C compared with the 0.97 to 1.57°C obtained with VALO at 3200 mW/cm^2^ (Group 2).

**Figure 7:**
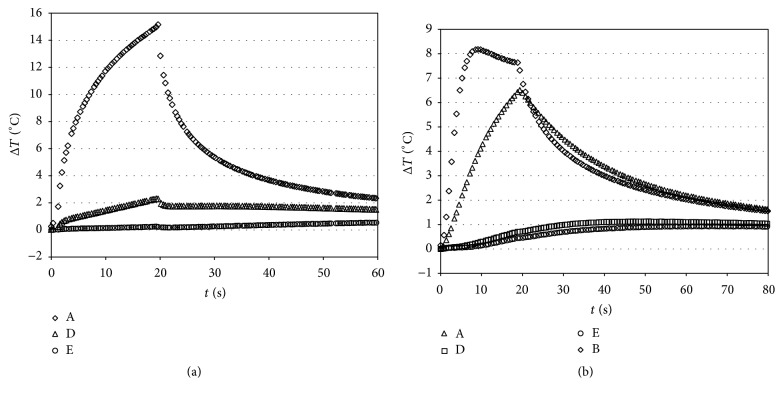
Characteristic temperature versus time curves caused by irradiation with Starlight PRO (Mectron) tested at a light intensity of 1000 (for 20 s) without (a) and with resin polymerization (b). A (1 mm from light source); B (bottom of the box); D (mesial); E (pulp chamber).

Figure 6 reports selected Δ*T* versus time curves for Starlight PRO (Mectron) tested at a light intensity of 1000 mW/cm^2^ (for 20 s). The slope of the curve changed due to temperature variations, showing a reduction in the power produced by the light-curing source. In fact, the temperature increase obtained with Starlight PRO was always lower compared with that obtained with VALO at 1000 mW/cm^2^ (Figure 7).

**Figure 8:**
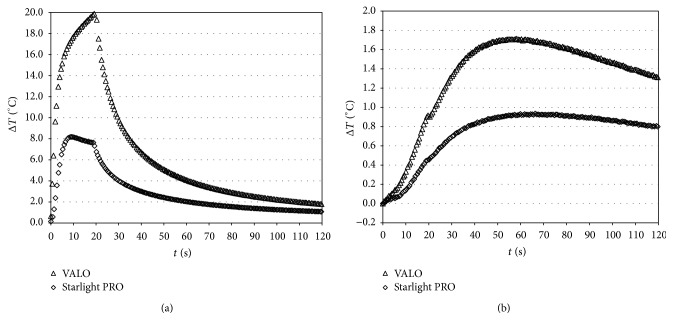
Characteristic Δ*T* (°C) versus time curves caused by irradiation with VALO (Ultradent) and Starlight PRO (Mectron) tested at a light intensity of 1000 (for 20 s) during composite polymerization obtained at the bottom of the restoration (a) and at pulp chamber (b).

The maximum temperature increase varied significantly depending on the two different light-curing units. With VALO, the temperature increase was continuous throughout the duration of exposure to the system, while in the case of Starlight PRO the temperature increase stopped after about 10 s and began to decrease, due to the characteristics of power delivery, resulting in the onset of cooling during the curing process (Figure 8).

## 4. Discussion

One way to compensate for reduced light source intensity with distance would be to increase the exposure time, which would maintain a constant level of total energy supplied to the resin composite [5]. The difference in degree of conversion obtained with increasing radiant exposure is due to an increase in free radical concentration and is also influenced by the effect of temperature on the mobility of the reactive species [20]. Conversely, the main thrust has been the development of lights that would result in faster cure of resin composites and generate less heat. The LED curing units that have been introduced are known to have an emitting radiation with a narrow spectral range (peak around 470 nm), which matches the optimum wavelength for the activation of the camphorquinone photoinitiator. Curing times longer than those recommended by the manufacturer improve polymerization and decrease the permeability of simplified dentin adhesives. In contrast, light-curing units can cause a temperature increase. In fact, a temperature increase of 5.5°C within the pulp chamber would lead to irreversible pulpal damage [21]. However, it is questionable as to whether the values obtained in monkeys are also valid for humans. In fact, Jakubinek et al. [22] have shown that the pulpal tissues could tolerate a temperature increase >5.5°C without damage. Thermal transfer to pulp is influenced by material shade, thickness, composition, porosity, curing times, and residual dentin thickness. The irradiance of 0.5 mm thick human dentin discs with a QTH (6.4°C) in comparison with a LED (3.4°C) curing unit promoted a higher temperature increase, which propagated through the dentin, negatively affecting the metabolism of the underlying cultured odontoblast-like cells [23].

The objective of the present study was to measure the temperature increase during a microhybrid composite polymerization by utilizing two different light-emitting-diode curing units (LED).

Many authors have quantified the amount of heat generated in resin-containing material during visible-light curing. The maximum temperature increases measured by thermographic investigation were 43.1°C for flowable composite and 32.8°C for conventional composite [24]. The temperature increase with LED lamps varies from 41°C to 53°C [11].

Measurements made when the lamp was used without curing the composite allowed for verification of the mode of transmission of heat from the surface to other parts of the tooth. Based on the temperature at the start of light exposure, a delay in pulp chamber temperature increase was observed; furthermore, a reduction of the temperature increase in the position not directly irradiated by the lamp was noticed (sections  D and E of Figure 4).

The temperature increase caused by light duration was proportional to the time of lamp ignition (20 s and 3 s) as can be easily seen by the comparison between graphs of Figure 5(a) versus Figure 5(b). The trend obtained by this measurement is of the first order; this behavior was evident in position A. A prolonged exposure for 20 s at 1000 mW/cm^2^ resulted in higher elevations of temperature with respect to light intensity of 3200 mW/cm^2^ for 3 s, although the power was lower. The exposure to the light source Starlight PRO for another 20 s (in addition to the first one) showed a temperature increase in the pulp chamber higher with respect to that observed with VALO at 1000 mW/cm^2^. This confirms the results of previous studies reporting that overcuring is potentially dangerous and points to the importance of developing curing protocols for specific combinations of lights and composites to ensure that restorations are fully cured and that the temperature is minimized [22]. Temperature increases in all the light-curing units were well within the normal range of pulpal physiology.

The temperature increase measured at the bottom of a box could be important for dentin properties, since enamel and dentin have different thermal and mechanical properties, and the thermal diffusivity and Young's modulus of enamel are approximately 2.5 and 4 times larger, respectively, than those of dentin. The difference in these properties may result in thermal stress and cracking within the tooth when subjected to a thermal stimulus.

One factor determining intrapulpal temperature increase is remaining dentin thickness. In the present study, the dentin remaining between the pulpal floor and the pulp chamber was not sufficiently isolated from light curing. In fact, conventional wisdom has established the use of lining materials to afford protection to the pulp and insulate the pulp from the extremes of thermal stimuli, particularly after restoration procedures. Dentin-based resins and resin-modified glass polyalkenoate are considered the most efficient thermal insulators [25].

In addition, from Figure 6 it can be seen that the first part of heating in the pulp chamber is due to transparency of the tissue to the radiation, then the contribute of the heat released during polymerization starts. This phenomenon is clearly evident at 3200 mW/cm^2^ where the length of radiation is short and the overlapping with the heat propagation is limited.

## 5. Conclusions

Intrapulpal peak temperature during resin curing is a function of the rate of polymerization and is due to the exothermic polymerization reaction, the energy from the light unit, and time of exposure. Temperature measurement showed also that there is a contribution of the direct radiation that reaches the pulp chamber and this contribution is higher with high time application (20 s versus 3 s).

Differences in temperature increases during composite polymerization were found between the two LED lights tested, regardless of the sites selected.

The results indicated that intrapulpal temperatures increase during composite photocuring, confirming that thermal transfer to the pulp is affected by the remaining dentin thickness, even if these increments are always lower than 4.74°C.

The hypothesis was accepted but further studies are needed to clarify* in vivo* the clinical relevance of temperature increase during light-activated polymerization.

## Figures and Tables

**Table 1:** Preliminary lamps test, nominal power, and real power measured with a bolometer.

Time (s)	Mectron Starlight PRO				VALO Ultradent			
	Nominal power (mW/cm^2^)	Real power (mW/cm^2^)	Nominal energy (J)	Real energy (J)	Nominal power (mW/cm^2^)	Real power (mW/cm^2^)	Nominal energy (J)	Real energy (J)
3	—	—	—	—	3200	1600	8.31	4.15
20	1000	750	10.05	7.54	1000	613	17.32	10.61

**Table 2:** Paired samples statistics (results for *t*-tests, ANOVA, and other comparison methods). Pre: prepolymerization. Intra: intrapolymerization. Temperature rise at different thermocouple position (ABCDE).

	Groups	Mean	Std. Er.	Std. Dev	*t*	dof	Wilcoxon test	Kruskal-Wallis test	One-way ANOVA *F* test	Pearson *χ* ^2^ test
Pre-A	*VALO 1000*	3.21	0.17	0.83	12.21	27.53	5.81 (0.0000)	33.80 (0.0001)	149.08 (0.0000)	46.00 (0.000)
	*VALO 3200*	0.97	0.06	0.30						
Pre-D	*VALO 1000*	2.66	0.09	0.43	3.26	26.96	3.20 (0.0014)	10.23 (0.0014)	10.60 (0.0022)	14.70 (0.000)
	*VALO 3200*	1.74	0.27	1.28						
Pre-E	*VALO 1000*	2.75	0.19	0.91	4.96	40.63	3.81 (0.0001)	14.54 (0.0001)	24.58 (0.0000)	7.04 (0.008)
	*VALO 3200*	1.57	0.14	0.68						
Pre-C	*VALO 1000*	21.78	0.98	4.69	−9.08	43.90	−5.26 (0.0000)	27.69 (0.0001)	82.42 (0.0000)	31.39 (0.000)
	*VALO 3200*	34.66	1.03	4.93						
Intra-C	*VALO 1000*	3.97	0.23	1.12	3.33	42.08	2.41 (0.0161)	5.789 (0.0161)	11.10 (0.0018)	4.26 (0.039)
	*VALO 3200*	2.73	0.29	1.39						
Intra-D	*VALO 1000*	2.55	0.14	0.67	8.51	37.90	4.92 (0.0000)	24.23 (0.0001)	72.46 (0.0000)	25.13 (0.000)
	*VALO 3200*	1.13	0.09	0.44						
Intra-E	*VALO 1000*	3.34	0.29	1.39	5.77	33.46	4.27 (0.0000)	18.26 (0.0001)	33.28 (0.0000)	10.52 (0.001)
	*VALO 3200*	1.45	0.15	0.74						
Intra-B	*VALO 1000*	18.89	0.92	4.43	−1.95	43.90	−1.75 (0.0807)	3.05 (0.0807)	3.79 (0.0581)	0.09 (0.76)
	*VALO 3200*	21.37	0.88	4.22						

Notes: unequal variances assumed, after some checks. After ANOVA, Bonferroni multiple-comparison test has been performed.

**Table 3:** Paired samples statistics (results for *t*-tests, ANOVA, and other comparison methods). Pre: prepolymerization. Intra: intrapolymerization. Post: postpolymerization. Temperature rise at different thermocouple position (ABCDE).

	Groups	Mean	Std. Er.	Std. Dev	*t*	dof	Wilcoxon test	Kruskal-Wallis test	One-way ANOVA *F* test	Pearson *χ* ^2^ test
Pre-A	*Mectron 1000*	2.09	0.11	0.51	−5.50	36.66	−4.12 (0.0000)	16.98 (0.0001)	30.29 (0.0000)	8.71 (0.003)
	*VALO 1000*	3.21	0.17	0.83						
Pre-D	*Mectron 1000*	1.78	0.09	0.41	−7.10	43.84	−4.76 (0.0000)	22.63 (0.0001)	50.44 (0.0000)	19.57 (0.000)
	*VALO 1000*	2.66	0.09	0.43						
Pre-E	*Mectron 1000*	1.22	0.07	0.32	−7.57	27.30	−4.86 (0.0000)	23.58 (0.0001)	57.29 (0.0000)	25.13 (0.000)
	*VALO 1000*	2.75	0.19	0.91						
Pre-C	*Mectron 1000*	17.88	0.49	2.36	−3.55	32.45	−2.70 (0.0069)	7.30 (0.0069)	12.63 (0.0009)	4.26 (0.039)
	*VALO 1000*	21.78	0.98	4.69						
Intra-C	*Mectron 1000*	2.45	0.08	0.38	−6.16	27.06	−5.38 (0.0000)	28.98 (0.0001)	37.91 (0.0000)	38.35 (0.000)
	*VALO 1000*	3.97	0.23	1.12						
Intra-D	*Mectron 1000*	1.84	0.07	0.34	−4.54	32.50	−3.68 (0.0002)	13.55 (0.0002)	20.57 (0.0000)	17.08 (0.000)
	*VALO 1000*	2.55	0.14	0.67						
Intra-E	*Mectron 1000*	2.21	0.15	0.73	−3.46	33.20	−2.83 (0.0046)	8.03 (0.0046)	11.98 (0.0012)	4.26 (0.039)
	*VALO 1000*	3.34	0.29	1.39						
Intra-B	*Mectron 1000*	15.77	0.69	3.30	−2.71	40.68	−2.47 (0.0134)	6.11 (0.0134)	7.35 (0.0095)	2.17 (0.140)
	*VALO 1000*	18.89	0.92	4.43						
Post-C	*Mectron 1000*	1.56	0.08	0.36	−11.62	43.40	−5.75 (0.0000)	33.01 (0.0001)	135.13 (0.0000)	38.35 (0.000)
	*VALO 1000*	2.74	0.07	0.32						
Post-D	*Mectron 1000*	1.57	0.07	0.34	−6.53	43.89	−4.60 (0.0000)	21.19 (0.0001)	42.66 (0.0000)	19.57 (0.000)
	*VALO 1000*	2.20	0.07	0.32						
Post-E	*Mectron 1000*	2.30	0.07	0.33	−6.11	32.43	−4.58 (0.0000)	20.99 (0.0001)	37.35 (0.0000)	25.13 (0.000)
	*VALO 1000*	3.24	0.14	0.66						
Post-B	*Mectron 1000*	13.77	0.51	2.46	0.68	42.81	0.59 (0.5530)	0.35 (0.5530)	0.46 (0.5028)	0.09 (0.768)
	*VALO 1000*	13.31	0.43	2.08						

Notes: unequal variances assumed, after some checks. After ANOVA, Bonferroni multiple-comparison test has been performed.
